# Dose–response of rPMS for upper Limb hemiparesis after stroke

**DOI:** 10.1097/MD.0000000000020752

**Published:** 2020-06-12

**Authors:** Shoji Kinoshita, Kumi Ikeda, Shinji Yasuno, Sho Takahashi, Naoki Yamada, Yumi Okuyama, Nobuyuki Sasaki, Takuya Hada, Chiaki Kuriyama, Shin Suzuki, Midori Hama, Naoto Ozaki, Shu Watanabe, Masahiro Abo

**Affiliations:** a Department of Rehabilitation Medicine; b Clinical Research Support Center, The Jikei University School of Medicine, Minato-Ku, Tokyo, Japan.

**Keywords:** clinical trial, neurophysiology, rehabilitation medicine, stroke, stroke medicine

## Abstract

**Introduction:**

Repetitive peripheral magnetic stimulation (rPMS) therapy is an innovative and minimally invasive neurorehabilitative technique and has been shown to facilitate neural plasticity. However, there is at present no research that clarifies the dose–response of rPMS therapy on the recovery of upper limb hemiparesis after stroke. This trial aims to clarify the dose–response of rPMS therapy combined with intensive occupational therapy (OT) for chronic stroke patients with moderate to severe upper limb hemiparesis.

**Methods and analysis:**

This multicenter, prospective, assessor-blinded, randomized controlled study with 3 parallel groups will be conducted from January 20, 2020 to September 30, 2022. Fifty patients will be randomly assigned in a ratio of 1:2:2 to the control group, the group receiving daily 2400 pulses of rPMS, or the group receiving daily 4800 pulses of rPMS, respectively. From the day after admission (Day 1), rPMS therapy and intensive OT will be initiated. The primary outcome is the change in the motor function of the affected upper extremity (Fugl-Meyer Assessment) between the time of admission (Day 0) and the day after 2 weeks of treatment (Day 14). Secondary outcomes will include the changes in spasticity, active range of motion, motor evoked potential, and activity of daily living.

**Ethics and dissemination:**

The study was approved by the Jikei University Certified Review Board for all institutions (reference number: JKI19–020). Results of the primary and secondary outcomes will be published in a peer-reviewed journal and presented at international congresses. The results will also be disseminated to patients.

**Trial registration number:**

jRCTs032190191.

## 1 Introduction

Stroke is a major global health care problem, and rehabilitation is a major component of clinical management. Among post-stroke sequelae, hand and arm impairment often persists and has a particularly strong impact on a person's activities of daily living (ADL) and quality of life (QOL).^[1]^ The primary goal of stroke rehabilitation is to achieve higher levels of motor function recovery of the affected upper limb, leading to better ADL and QOL.

Recent advances in clinical neurophysiology have led to the development of novel neurorehabilitative techniques for facilitating neuroplasticity and recovery of affected upper limb motor function. Neuromuscular electrical stimulation (NMES) has been developed and validated for improving both the motor function of the affected upper limb and ADL in stroke patients.^[2,3]^ However, NMES has some adverse effects, including pain, dermatitis, and skin burns. Furthermore, the depth of stimulation produced by NMES is very shallow, which does not result in sufficient stimulation of the deep muscles, such as the supraspinatus muscle. In contrast, the beneficial effect of repetitive transcranial magnetic stimulation (rTMS) therapy in patients with post-stroke upper limb hemiparesis has been previously reported.^[4,5]^ However, the beneficial effect of rTMS has only been demonstrated for stroke patients with mild hemiparesis. In addition, the clinical application of rTMS is limited to patients without a history of epilepsy. There is also a possibility that rTMS might lead to the development of epilepsy.

Because of these limitations, this study focuses on the use of repetitive peripheral magnetic stimulation (rPMS) therapy, which is a relatively safe and minimally invasive neurorehabilitative technique. rPMS therapy involves generating a magnetic field in the vertical direction by passing an electric current through a magnetic coil and selectively stimulating a nerve or muscle. The principle behind rPMS is similar to that of NMES; however, rPMS can penetrate the deeper muscle layers and is nearly painless, with virtually no side effects. The repetitive contraction–relaxation cycles produced by rPMS have been shown to both enhance proprioceptive input from the affected extremity, and to increase neuroplasticity.^[6–8]^ Several studies have reported the clinical utility of rPMS therapy in acquired brain injury patients with dysphagia,^[9]^ gait disturbance^[10]^ leg paralysis after stroke,^[11]^ and constipation.^[12]^

Two clinical trials evaluating the effect of rPMS on upper limb hemiparesis have previously been conducted in stroke patients. In the randomized controlled trial (RCT) conducted by Krewer et al, rPMS therapy for acquired brain injury patients with upper limb paralysis had a significantly greater effect on spasticity compared with the sham stimulation group; however, there was no significant effect of rPMS on the motor function of the affected upper limb.^[13]^ The rehabilitation treatment in this study involved passive range of motion (ROM) movements and stretching, and did not include the active training required to facilitate voluntary movements of the hand and arm. We believe that a protocol of rPMS combined with intensive occupational therapy (OT) will provide a therapeutic effect in the improvement of upper limb function. Although a previous case–control study reporting the usefulness of rPMS therapy for patients with subacute stroke^[14]^ has been conducted; there have been no randomized controlled trials that have reported the therapeutic effects of rPMS therapy in chronic stroke patients.

In addition, there is no established protocol that exists for rPMS therapy, and the most effective stimulation count of rPMS per day for functional recovery from stroke is still unknown. With regard to the stimulation frequency, previous neurophysiological and clinical studies have shown that 20 to 30 Hz of rPMS therapy is effective.^[9–10,14–17]^ On the contrary, there are various reports on the total number of rPMS stimuli per day to produce a treatment effect, ranging from 1200 to 16,000.^[9–13]^ In a case–control study focusing on upper limb paralysis, the usefulness of rPMS therapy with a daily total stimulus delivery of 5000 was reported.^[14]^ However, there has been no data reported regarding the dose–response of rPMS therapy.

Therefore, we decided to examine the dose–response of daily total stimulus delivery in rPMS therapy in the treatment of upper limb paralysis secondary to stroke. The purpose of this RCT is to clarify this dose–response when combined with intensive OT in chronic stroke patients with moderate to severe upper limb hemiparesis, comparing three parallel groups. In addition, we aimed to assess the safety of rPMS therapy by comparing the incident rate of adverse events (AE) among groups.

## 2 Methods and analysis

### 2.1 Trial design

This study is a multicenter, prospective, assessor-blinded, dose–response RCT study with 3 parallel groups. It will be conducted from January 20, 2020 to September 30, 2022. This trial aims to clarify the dose–response of rPMS therapy when combined with intensive OT in chronic stroke patients with moderate to severe upper limb hemiparesis. Two hospitals (the Jikei University Hospital and the Jikei University Daisan Hospital) have been registered as study sites.

### 2.2 Patient and public involvement

Neither the patients, nor the public were directly involved in the study design, patient recruitment, and conduct of the study. The obtained results will contribute to better clinical outcomes for stroke patients with upper limb hemiparesis.

### 2.3 Sample selection

Inclusion criteria are:

1. Stroke patients with upper limb hemiparesis between the age of 18 and 80 years;
2. More than or equal to 3 months passing since the occurrence of stroke;
3. Patients with a Brunnstrom Stage of 3 to 4 for the upper limb;
4. Patients with a generally stable condition;
5. Patients with a lack of cognitive impairment, and a good understanding of the study plan;
6. Patients who are able to give written informed consent of their own free will;
7. Patients of female or male sex;
8. Patients who are able to adhere to the study protocol, including hospitalization and outpatient visits; and
9. Patients who have previously been treated with botulinum toxin (BTX) therapy for upper limb paresis.

In this study, we only included patients who had received BTX therapy for their affected upper limb, as this method has been validated for reducing spasticity.^[18]^ In a previous RCT, rPMS therapy was shown to improve spasticity,^[13]^ thus indicating that administration of rPMS may be linked to the improvement of motor function and the reduction of spasticity. Although it might be desirable to include patients without the experience of BTX therapy, it should be considered that recruitment of these patients is difficult, as almost all patients with severe upper limb hemiparesis, who have had spasticity in their affected limb, have been treated with BTX. Based on this, and to ensure the treatment procedure for spasticity is uniform, we decided to recruit stroke patients who have previously received BTX therapy.

Exclusion criteria are:

1. Patients with a self-contained medical implant (pacemaker, cochlear implant, and so on);
2. Patients with severe heart disease;
3. Patients with metal implants near the rPMS stimulation site;
4. Patients with deep-vein thrombosis near the rPMS stimulation site;
5. Patients with infection (such as acute cellulitis, and so on) near the rPMS stimulation site;
6. Patients who are, or who may be, pregnant;
7. Patients who have unstable diseases that should be immediately treated (such as acute heart failure, acute kidney injury, severe diabetes, infection, and so on);
8. Patients who have received BTX therapy within 2 weeks of admission, as the drug efficacy of BTX is known to gradually develop up to 2 weeks after the injection^[19]^; and
9. Patients who are otherwise considered ineligible for the study according to the researchers.

### 2.4 Study procedure

**Figure 1:**
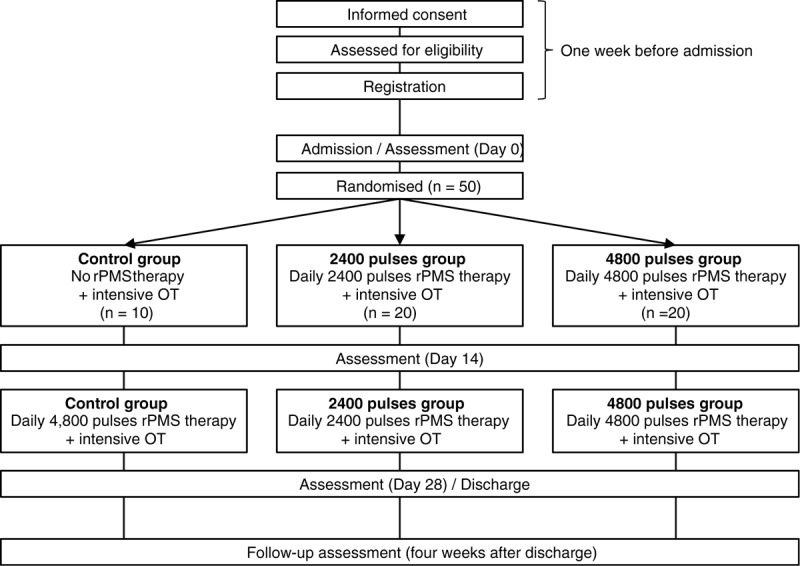
Study flow chart.

Eligible patients who provide written informed consent will be randomly assigned at a ratio of 1:2:2 to the control group, the group receiving 2400 daily pulses of rPMS (2400 pulses group), or the group receiving 4800 daily pulses of rPMS (4800 pulses group), respectively. Patients will be randomly assigned using a computer-generated list of random numbers using blocked randomization, and stratified by the Fugl Meyer Assessment (FMA) (<20 or ≥20) and age (<65 or ≥65 years’ old). The study researcher will report to the allocator by phone, and the assignment will then be reported to the investigator. The block sizes will not be disclosed to ensure allocation concealment. rPMS therapy and intensive OT will be initiated from the day after admission (Day 1). The evaluation will be conducted on Day 14, after 2 weeks of therapy. After the evaluation, the therapy will be repeated for another 2 weeks (Day 15–28). For the control group, 4800 pulses of rPMS therapy will be performed for relief measures after the evaluation (Day 14). Another evaluation will be conducted 2 weeks after therapy (Day 28). Patients will be discharged on Day 28, after a total of 4 weeks of admission. In addition, the immediate effect of rPMS therapy will be assessed at the time of admission and the first session of rPMS therapy. We will check the long-term effect and safety of the rPMS therapy four weeks after discharge. The study flow chart is shown in Figure 1.

The investigators and participating patients will not be blinded to the group assignment, although the blinding of outcome assessors will be ensured. The evaluating occupational therapists will be instructed to refrain from attempting to ascertain the group to which the patient has been allocated, and the patients will be instructed not to discuss their treatment allocation to any of the clinicians without the physiatrists.

### 2.5 Study interventions

**Figure 2:**
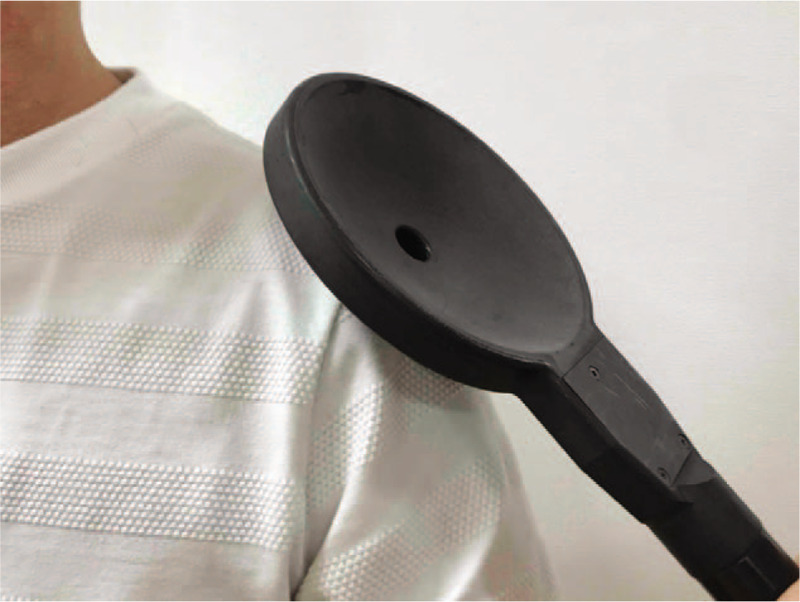
Photograph illustrating the application of repetitive peripheral magnetic stimulation (rPMS) therapy using a parabolic coil. Sufficient stimulation of the deep layer of muscles is possible by applying the rPMS even through the clothes.

Our study will consist of 2 interventions: rPMS therapy and intensive OT. rPMS therapy will be applied using the Mag Pro R30 stimulator with a 140 mm parabolic coil (Magventure, Denmark). A stimulation coil will be percutaneously placed on the target muscles (Fig. 2). Each train of rPMS stimuli will be applied at 20 Hz for 3 seconds followed by a 27 second rest interval. Eighty such trains of rPMS stimuli will be applied as the daily 4800 pulses of rPMS therapy, and 40 such trains of rPMS stimuli will be applied as the daily 2400 pulses of rPMS, in the respective treatment groups. As the previously mentioned case–control study has shown the usefulness of 4800 pulses of rPMS therapy in the recovery of upper limb motor function in patients with subacute stroke,^[12]^ we decided to clarify the response to this 4800 pulses dose, to half of this dose (2400 daily stimuli of rPMS), and to no treatment. The intensity of rPMS will be individually set to 10% above the minimal level that evoked a joint movement when the patient was at rest.^[10,13]^ The participating physiatrists will select the rPMS stimulation site. In this study, it is required that more than half of rPMS stimulation are performed on the proximal muscles of the upper limb, such as the deltoid, triceps brachii, and supraspinatus. More than 1200 pulses of rPMS in a single stimulation site will be prohibited. The dose will be decreased or the rPMS will be discontinued if the patient requests, or if side effects of rPMS are observed. Once any side effects have improved, the investigator will be allowed to discontinue or resume treatment with modifications, according to the protocol.

A single session of intensive OT will consist of 60 minutes of face-to-face therapy, provided by an experienced occupational therapist, followed by 60 minutes of self-training. Two sessions of rehabilitative training will be provided during hospitalization for a daily total of 240 minutes. The main objectives of the training sessions are to improve the upper limb hemiparesis and to increase the frequency of use in ADLs of the hand and upper limb on the paralyzed side. To address the hemiparesis of the upper limb, we will provide muscle-strengthening and range of motion (ROM) exercises, based on an assessment of the alignment of the trunk and the proximal portion of the upper limb, and an assessment of the muscle tone. These exercises are aimed at improving support and increasing the ROM of the proximal portion of the upper limb. After performing a similar assessment of the distal portion, we will aim to change from what is presently a cooperative movement of multiple fingers, such as grip and release, to isolated movements of a finger. In addition, we will provide repeated combined motion training of both hands using objects such as those used in ADL-exercises, based on the information provided by the patient or family on the first day of the hospitalization. The self-training portion will be performed in another quiet room without any supervisors after the one-on-one with the OT. This self-training programme will be provided by the occupational therapists based on the contents of individualized training, using written instruction.

NMES, rTMS, transcranial direct current stimulation, and robotic rehabilitation will be prohibited during the study period, as these therapies may affect the efficacy and safety evaluation of the study treatment. In addition, new administration of muscle relaxant agents will be prohibited. However, if the study patient has been already taking muscle relaxants, the dosage will not be changed during the study period. Should the patients experience any harm from the trial, we will then provide the appropriate medical care for each condition.

### 2.6 Outcome measures

The evaluations will be conducted at 1 week before the admission; on the day of admission (Day 0); 1 day after admission (Day 1); 2 weeks after admission (Day 14); 4 weeks after admission (Day 28); at the point of discontinuation; and 4 weeks after discharge. The assessment schedule is shown in Table 1.

**Table 1:**
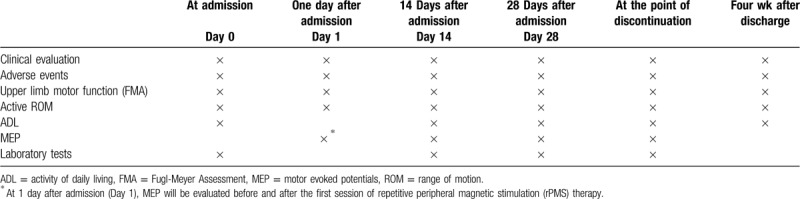
Assessment schedule during the study period.

### 2.7 Primary outcome

The primary outcome of the study is the difference in the upper limb motor function (FMA) between Day 0 and Day 14. The FMA is a performance-based quantitative measure that assesses various impairments in post-stroke patients.^[20]^ The upper limb motor function section in the FMA consists of 33 items. Each item is rated on a 3-point scale of 0 points (cannot perform), 1 point (can partially perform), and 2 points (can fully perform). This scoring allows for a maximum motor performance score of 66 for the upper limb. Furthermore, the FMA is comprised of the following 4 categories of evaluation: a category for shoulder/elbow/forearm function; a category for wrist function; a category for hand function; and a category for coordination/speed.

### 2.8 Secondary outcomes

The secondary outcomes in the present study are differences in spasticity, active ROM, motor evoked potential (MEP), and ADL during the study period. Differences in FMA scores between Day 0 and Day 28 and between Day 14 and Day 28 will be also analyzed. We will also evaluate the immediate changes of FMA score, active ROM, and MEP after the first session of rPMS therapy. Differences in FMA score, spasticity, active ROM, and ADL between day 28 and 4 weeks after discharge will also be analyzed.

Spasticity will be evaluated by using the modified Ashworth Scale (MAS), which is a 6-grade semiquantitative measurement used to assess the severity of spasticity.^[21]^ MAS scores for the shoulder, wrist, and finger flexors/extensors will be evaluated for each patient.

Active ROM goniometry will be evaluated for shoulder flexion/extension and abduction/adduction, elbow flexion/extension, hand flexion/extension, and finger flexion/extension. Subjects will be seated in a straight-backed chair and be must able to maintain this seated position for the duration of testing. Subjects will be instructed to make different movements of the joint while keeping the other upper extremity joints still, all the while seated and with the upper extremity hanging down.^[22]^

Moreover, to obtain electrophysiological measures of corticospinal integrity, the MEP amplitude induced by a single-pulse transcranial magnetic stimulation to the ipsilesional primary motor cortex will be recorded using a MagPro R30 magnetic stimulator and a figure-of-eight coil with a 70 mm radius (Magventure; Denmark). Because affected-arm MEPs could not be elicited at rest, we will determine the optimal coil position where a MEP will be elicited during the voluntary activation of triceps brachii.^[23]^ Eight stimulations will be performed with intensity set at 80% of maximum stimulator output and the averaged amplitude will be considered for statistical analysis.

The Functional Independence Measure (FIM) will be used for evaluating ADLs. FIM is a standardized and widely used tool for the assessment of ADLs, and its reliability and validity in the rehabilitation setting have been confirmed previously.^[24]^ The FIM includes 18 items rated on a 7-point scale, where the subtotal-summed scores of motor subscales (motor FIM) are used to quantify functional independence.

To assess any muscular injury induced by rPMS therapy, laboratory tests for serum aspartate aminotransferase (AST), lactate dehydrogenase (LDH), and creatine kinase (CK) will be serially checked.

### 2.9 Data management and monitoring

The research investigator will note the outcome data for each patient at each assessment point, as well as any deviation from the protocol or AE, on the case report form (CRF). The CRF data associated with the outcome indicator will undergo double verification by 2 researchers, and the researcher will be responsible for the accuracy of data entry for each study institution. After verification, the CRF will be sent to the data manager. All hard copies of study-related information will be securely stored in a location with restricted access. The outcome data will be stored without the patients’ personal names; instead, a study-specific patient identifier based on the correspondence table will be used.

The data manager of this study will also monitor protocol compliance, safety, and on-schedule study progress. This is a study with a relatively small sample size and limited number of participating institutions; therefore a data monitoring committee will not be established. An audit is not scheduled to be performed within the study but will be done if necessary.

The monitor of this study will confirm that the study is being conducted in compliance with the latest research protocol and regulatory requirements throughout the study period, according to the monitoring procedures that was approved by the Certified Review Board.

### 2.10 Sample size estimation

The target sample size for this randomized trial is 50. This number is based on the results of a previous case–control study in subacute stroke by Yang et al, which compared the improvement of FMA changes between patients who received both rPMS therapy in the shoulder muscles and rehabilitation therapy (treatment group), and the patients who received both NMES therapy and rehabilitation (control group). This study revealed that the FMA score in the rPMS and control groups improved by 11.47 ± 2.72 and 5.40 ± 1.80 (mean ± standard deviation), respectively.^[14]^ Occupational therapy alone has been shown to improve FMA scores by 3.4 in the chronic phase of stroke.^[25]^ Based on these reports, we predicted that a therapeutic effect of the intervention for chronic stroke may occur at a 65% of that for subacute stroke. In addition, we predicted that the therapeutic effect in the group receiving 2400 pulses of rPMS will occur at 50% of those receiving 4800 rPMS in therapy. The estimated changes in the FMA scores of the control group, 2400 pulses group, and 4800 pulses group are therefore 3.4, 5.4, and 7.4, respectively, and the assumed standard deviation is 3. A sample size of 8 patients in the control group, 16 patients in the 2400 pulses group, and 16 in the 4800 pulses group will provide an 80% power to detect differences of the change in FMA among the groups, using the maximum contrast method with contrast coefficients (−1, 0, 1), (−1, −1, 2), (−2, 1, 1) at a 5% level of significance (2-sided). A dropout rate of 20% is allowed. A total sample size of 50 patients is hence required for the trial.

### 2.11 Statistical analysis

The analyses of the primary and secondary efficacy outcomes will be performed using the Full Analysis Set. Safety analysis will be conducted in the safety analysis population. For baseline characteristics, summary statistics will comprise frequencies and proportions for categorical variables, and means and SDs for continuous variables. The patient characteristics will be compared using Pearson *χ*^2^ test or Fisher exact test for categorical variables, and 1-way analysis of variance (ANOVA) for continuous variables.

For the primary analysis, to evaluate the responsiveness of FMA change to the daily total stimulus count of rPMS in chronic stroke patients with upper limb paralysis, the maximum contrast method will be performed with contrast coefficients (−1, 0, 1), (−1, −1, 2), and (−2, 1, 1). A change in the FMA score between Day 0 and Day 14, with the corresponding 95% confidence interval (CI), will be estimated for each group.

For the secondary analysis, Pearson *χ*^2^ test will be performed for categorical variables, and 1-way ANOVA for continuous variables. The significance level of the 2-sided hypothesis test is 5%, and the CI is a 2-sided 95% CI.

For the safety analysis, the frequencies of AEs will be compared using Pearson *χ*^2^ test or Fisher exact test, as appropriate.

All comparisons have been planned, and all *P* values will be 2-sided. *P* values < .05 will be considered statistically significant. All statistical analyses will be performed using SAS software, V.9.4 (SAS Institute, Cary, NC). The plan for statistical analysis was developed by the chief investigator and statisticians, and will be finalized before the database lock.

### 2.12 Ethics and dissemination

This research will be carried out in accordance with the latest version of the Declaration of Helsinki and Clinical Trials Act in Japan. The study was approved on January 8, 2020 by the Jikei University Certified Review Board (reference number: JKI19–020) for all institutions. Verbal and written consent will be required from each patient. Results of the primary and secondary outcomes will be published in a peer-reviewed journal and presented at international congresses. The results will also be disseminated to patients. This study was registered as jRCTs032190191. The protocol version is V.1.3.

## 3 Discussion

This is the first clinical trial to investigate the dose–response of rPMS combined with intensive occupational therapy for the recovery of the motor function of the upper limb in patients with hemiparesis secondary to chronic stroke. The design of this study (assessor blinded, RCT) meets the highest level of evidence. However, this is a multicenter study with a relatively small number of participating institutions, and there is no blinding procedure for the physiatrists and patients. Once the most effective protocol of rPMS is validated through this study, a double-blind RCT with a large number of participating institutions should then be conducted to confirm its clinical effect.

In addition, this study will only include chronic stroke patients who have received BTX injections in the affected upper limb; this way, the treatment procedure for spasticity is uniform. However, because we will exclude patients in the acute or subacute phase or those without having received BTX therapy, we cannot generalize the study results.

## Acknowledgments

The authors acknowledge all the staff of the Department of Rehabilitation Medicine of the Jikei University School of Medicine for their support and assistance with the present study.

## Author contributions

**Conceptualization:** Shoji Kinoshita, Kumi Ikeda, Shinji Yasuno, Sho Takahashi, Masahiro Abo.

**Funding acquisition:** Shoji Kinoshita.

**Methodology:** Shoji Kinoshita, Kumi Ikeda, Shinji Yasuno, Sho Takahashi, Naoki Yamada, Yumi Okuyama, Takuya Hada, Chiaki Kuriyama, Shin Suzuki, Midori Hama, Naoto Ozaki, Masahiro Abo.

**Project administration:** Shoji Kinoshita, Shu Watanabe, Masahiro Abo.

**Supervision:** Shinji Yasuno, Naoki Yamada, Yumi Okuyama, Nobuyuki Sasaki, Takuya Hada, Shu Watanabe, Masahiro Abo.

**Writing – original draft:** Shoji Kinoshita, Kumi Ikeda.

**Writing – review & editing:** Shoji Kinoshita, Shinji Yasuno, Sho Takahashi, Naoki Yamada, Yumi Okuyama, Nobuyuki Sasaki, Takuya Hada, Chiaki Kuriyama, Shin Suzuki, Midori Hama, Naoto Ozaki, Shu Watanabe, Masahiro Abo.
